# Nerve reconstruction: A cohort study of 93 cases of global brachial plexus palsy

**DOI:** 10.4103/0019-5413.77136

**Published:** 2011

**Authors:** Anil Bhatia, Ashok K Shyam, Piyush Doshi, Vitrag Shah

**Affiliations:** Joshi Hospital and Sancheti Institute of Orthopaedics and Rehabilitation, Pune, Maharashtra; 1 Indian Orthopedic Research Group, Thane, Maharashtra, India; 2 Indian Orthopedic Research Group, Baroda, Gujarat, India; 3 Indian Orthopedic Research Group, Surat, Gujarat, India

**Keywords:** Brachial plexus injury, flail upper limb, nerve reconstruction

## Abstract

**Introduction:**

Brachial plexus injury leading to flail upper limb is one of the most disabling injuries. Neglect of the injury and delay in surgeries may preclude reinnervation of the paralysed muscles. Currently for such injuries nerve transfers are the preferred procedures. We here present a series of 93 cases of global brachial plexus palsy treated with nerve transfers.

**Materials and Methods:**

Ninety-three cases of global palsies out of 384 cases of brachial plexus injury operated by the senior surgeon (AB) were selected. Age varied from 4 to 51 years with 63 patients in 20 to 40 age group and all patients having a minimum follow up of at least 1 year post surgery ranging up to 130 months. The delay before surgery ranged from 15 days to 16 months (mean 3.2 months). The aim of the surgery was to restore the elbow flexion, shoulder abduction, triceps function and wrist and finger flexion in that order of priority. The major nerve transfers used were spinal accessory to suprascapular nerve, intercostal to musculocutaneous nerve and pectoral nerves, contralateral C7 to median and radial nerves. Nerve stumps were used whenever available (30 patients).

**Results:**

Recovery of ≥ grade 3 power was noted in biceps in 73% (68/93) of patients, shoulder abduction in 89% (43/49), pectoralis major in 100% (8/8). Recovery of grade 2 triceps power was seen in 80% (12/16) patients with nerve transfer to radial nerve. Derotation osteotomies of humerus (n=13) and wrist fusion (n=14) were the most common secondary procedures performed to facilitate alignment and movements of the affected limb. Better results were noted in 59 cases where direct nerve transfers were done (without nerve graft).

**Conclusion:**

Acceptable function (restoration of biceps power ≥3) can be obtained in more than two thirds (73%) of these global brachial plexus injuries by using the principles of early exploration and nerve transfer with rehabilitation.

## INTRODUCTION

Brachial plexus injuries affect the function of the upper limb by disconnection of the brain’s control. These lesions are on the rise because of efficient emergency life-saving services and increasing use of helmets by motorcyclists leading to better survival in cases of serious injuries.^1^–^3^ The extent of the deficit depends upon the level of the injury and the number of nerves affected. Closed traction is the usual mechanism and such injuries occur most often after falls from two-wheelers.^1^–^3^ Unfortunately, a majority of such lesions affect the supraclavicular part of the plexus (roots and trunks) and produce extensive paralyses of the upper limb.^4^–^6^ Around 45% of cases suffer global palsies with a complete loss of sensation and of motor function from the shoulder to the fingers. The victims of such accidents usually present with skeletal injuries affecting the same upper limb and/or the remaining three limbs as well as abdominal, chest, and head injuries.^1^ Fractures and other musculoskeletal injuries are primarily treated; however in many cases, the nerve injury is, sadly, neglected as many believe that the paralysis would recover spontaneously with time. Such expectant treatment would be justified only if clinical signs of recovery are visible in days and weeks after the accident (not months).^7^,^8^ However, most such injuries are severe and necessitate surgical exploration and reconstruction.^9^

**Figure 1:**
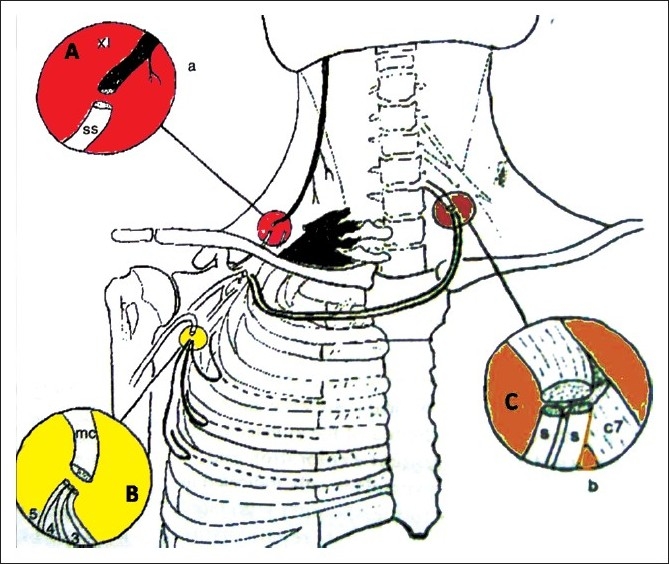
Diagrammatical representation of three most common nerve transfers used for flail upper limb secondary to brachial plexus injury. A- Spinal accessory to suprascapular nerve transfer, B-intercostals to musculocutaneous nerve transfer and C- Opposite C7 to musculocutaneous nerve transfer

Direct repair of the injured nerves is not possible as they are stretched and torn.^10^ Restoration of continuity involves placement of nerve grafts between the proximal stumps and the distal target nerves by microsurgical techniques. However, global palsies are usually associated with pre-ganglionic injuries, i.e. avulsions of the roots from the spinal cord.^11^ In the absence of available root stumps in the neck, the only recourse is to harness functioning nerves from outside the brachial plexus to serve as donors of growing axons that can, then, be connected to suitable target nerves (neurotizations or nerve transfers).^12^,^13^ The spinal accessory nerve (11^th^ cranial nerve that supplies the sternocleidomastoid and trapezius muscles), the phrenic nerve, and intercostal nerves are the common donors that have proved useful [Figure 1].^12^ In addition, Gu and his associates have shown that the C7 root of the opposite normal brachial plexus can be divided partly or completely and, then, bridged to the affected side using nerve grafts [Figure 1].^14^,^15^ In the absence of any function in the upper limb, one has to decide upon an order of priority for muscles to be reinnervated. It is universally accepted that restoration of elbow flexion should be the prime objective.^3^,^12^,^16^ Traditionally, shoulder stability and sensation with or without motor function in the median distribution have been the other usual aims of nerve reconstruction.^17^ This sequence has varied between various centers. In addition, several units prefer to perform staged procedures with two or more microsurgical operations. A single staged procedure is also acceptable.^18^ We prefer to utilize all available nerve transfers in the same operation. A delay of 6-8 months inevitably occurs before the results of such transfers are visible. In addition, none of these techniques has a 100% success rate. Experience in their use has shown that results are better and more consistent when the nerves are transferred to specific targets (motor branches to specific muscles) without interposition of nerve grafts.^19^ Limited availability of nerve transfers and grafts imposes rational use of these procedures to maximize return of function in these patients. We have selected global palsies as true representatives of the effectiveness of surgical treatment.

We hereby report a retrospective analysis of 93 cases of global brachial plexus palsies treated with nerve transfers over the past 13 years.

## MATERIALS AND METHODS

A series of 384 operated cases of post traumatic and obstetrical palsies brachial plexus injuries since April 1995 were reviewed. All the cases were operated by the senior author (AB). Of these, 177 (46%) were total palsies. Nine patients were lost to follow up within 3 months of surgery while in 75 cases follow up was inadequate for meaningful conclusions (follow up less than 1 year). Ninety-three cases with follow-up data of 12 months and more (12 to 130 months) were selected for the study. Only seven of these patients were female. The left side was affected in 49 patients. The age range was from 4 to 51 years. Sixty-three belonged to the age group of 20-40 years [Table 1]. The delay before nerve reconstruction surgery ranged from 15 days to 16 months (mean 3.2 months). Eighty-five of the 93 cases were operated upon within 6 months from the accident [Table 1] and remaining presented late due to neglect on part of patient and delay in referral from the primary treating surgeon.

**Table 1:** Distribution of cases according to age and delay before surgery

Age group in years	Number of cases	Pre-operative delay	Number of cases
1-10	5	15 days	1
11-20	15	1-3 months	53
21-30	42	3-6 months	32
31-40	21	6-12 months	4
41-50	9	>12 months	1
>50	1	Not recorded	2

Decision for surgery was made on clinical evaluation and any patient presenting with persistent deficit in the entire upper limb, absent function in the serratus anterior muscle, and presence of Horner sign was treated surgically. Suitable electro-diagnostic tests (EMG and nerve conduction studies) and, whenever possible, a cervical myelogram or an MRI were done to gain additional information regarding condition of nerve roots and extent of fibrosis These investigations were not used for diagnosis or decision of surgery which were based only on clinical examination.

### Surgical principles

The philosophy of nerve reconstruction in a flail upper limb involves an attempt to restore control in the maximum possible number of muscles and then improve the utility of the restored function by secondary surgeries. The most important muscle to be restored is the biceps. The basic surgery involves re-routing of healthy nerves to the distal stumps of injured nerves. If the distal stump is long enough, direct suturing of the transferred nerves can be achieved. In cases where stumps are small and transferred nerves do not reach the distal stump, nerve grafts are used to join the. The spinal accessory to musculocutaneous nerve transfer was preferred by the author in initial period (1995-1999). Subsequently the use of intercostals for the biceps helped free the spinal accessory for transfer to the suprascapular nerve and add shoulder abduction. In this context the transfer of the third intercostal nerve to the pectoral nerve has proved a most useful adjunct. For a brief period the senior author tried to use the contralateral C7 transfer for the biceps (six cases 2004-2006) so that intercostals could be transferred for the triceps; however the results were disappointing (biceps >grade 3 in two out of six patients). This forced the protocol to revert to intercostal transfer for biceps and contralateral C7 for median nerve and is followed till date.

**Figure 2:**
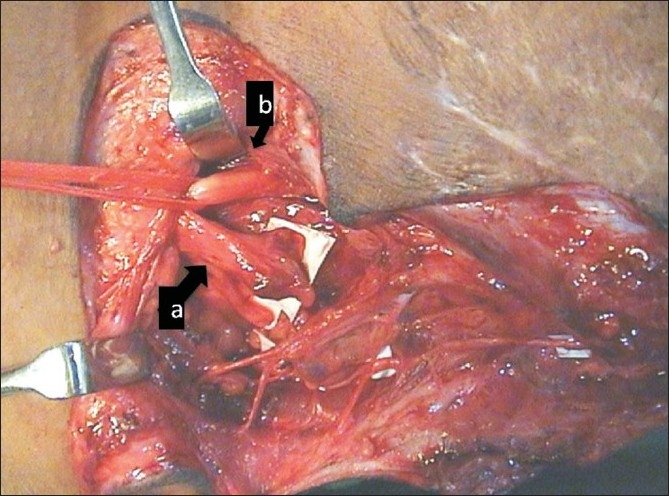
Exposure of the posterior triangle of the neck showing stumps of C5-C6 (a) and intact C7 (b)

**Figure 3:**
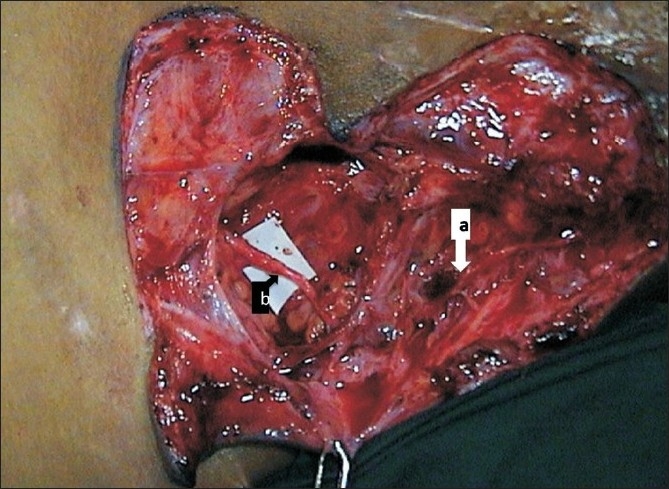
Intraoperative photograph showing nerve transfer between the spinal accessory nerve (a) and suprascapular nerve (b)

**Figure 4:**
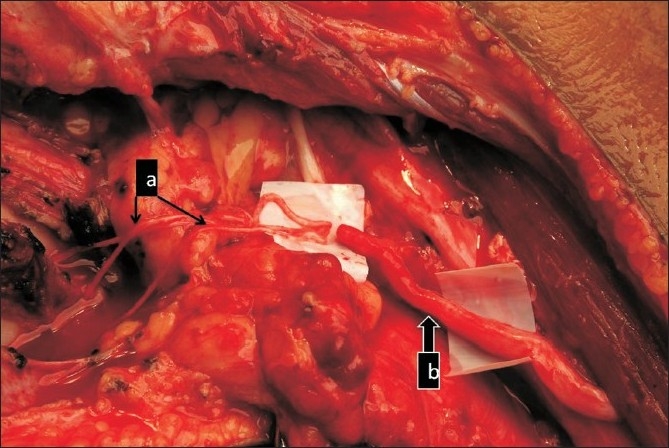
Photograph showing nerve transfer between intercostal nerves (a) to musculocutaneous nerve (b)

The surgical procedure involved systematic exploration of the posterior triangle to confirm the diagnosis and to look for available ruptured spinal nerves in the inter-scalene area [Figure 2]. The nerve stump was considered suitable for grafting if it was found uninjured at the intervertebral foramen with a neuroma distally and when stimulated produced contraction of serratus anterior muscle. The spinal accessory nerve was isolated at the entry to the trapezius [Figure 3]. An attempt was made to preserve the branches to the upper trapezius. The suprascapular nerve was isolated at its origin from the upper trunk in the same incision. The distal part of the plexus was exposed in the delto-pectoral region either via a delto-pectoral approach or by an axillary approach (the latter approach is currently preferred). Intercostal nerves were harvested by sub-periosteal separation of the intercostal muscles from the lower halves of the 3^rd^-5^th^ or 6^th^ ribs without osteotomies. Each intercostal nerve was traced from the posterior axillary line to the costo-chondral junction and divided anteriorly so that its length was sufficient to reach the target nerve in the axilla. Intercostal nerve transfers were used regularly from 1999, initially for the musculocutaneous nerve [Figure 4] and, later, for the radial nerve branch to the long head of the triceps. Since February 2007 a transfer of the third intercostal nerve to the pectoral nerve was also added.

When required the contra-lateral brachial plexus was exposed via a similar incision over the opposite posterior triangle. The upper trunk was identified by the proximity of the phrenic nerve and the origin of the suprascapular nerve and the C7 was exposed at the outer border of the scalenus anterior and traced distally. The posterior half of the C7 was separated under the microscope and divided distally.

**Figure 5:**
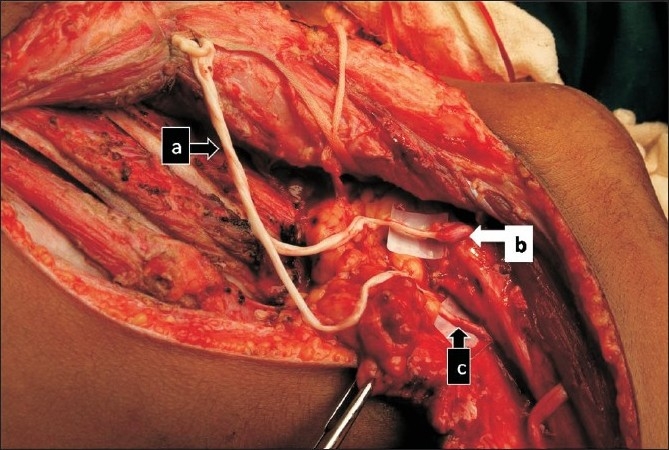
Intraoperative photograph showing use of sural nerve grafts (a) to bridge between the contralateral C7 and median nerve (b) and the radial nerve (c)

Sural nerves were harvested for use as nerve grafts whenever required [Figure 5]. Occassionally, the ipsilateral superficial radial nerve was taken. In three cases, a pedicled vascularized ulnar nerve graft was harvested (twice to the contralateral C7 and once to an ipsilateral C5 stump).

The spinal accessory nerve was transferred to the suprascapular nerve in 49 cases [Figure 3]. It was bridged to the musculocutaneous nerve using a nerve graft in 21 patients and, in three cases, to radial branch to the triceps. Three intercostal nerves were transferred to the musculocutaneous nerve in 59 patients [Figure 4]. Of these, a distal rupture of the musculocutaneous nerve (at the entry to the coraco-biceps) necessitated interposition of a nerve graft in three cases while a direct repair was possible in the remaining cases. This transfer was employed for the triceps in 15 cases (usually 2 intercostals). In addition, the third intercostal nerve was transferred to the pectoral nerve in 8 of these 93 patients.

Proximal nerve stumps that could be utilized as a source for axons were joined to the distal target nerves using nerve grafts. Such stumps were found in 30 cases- one stump in 26 patients, two stumps in 3 patients and all five roots were found ruptured in the interscalene area in 1 patient. Nerve grafting to the musculocutaneous nerve was performed in seven patients and other target nerves are listed in Table 2.

The contralateral C7 transfer was utilized in 32 cases. In six patients, the C7 was bridged to the lateral cord or the musculocutaneous nerve. In 24 patients, it was connected to the median nerve [Figure 5] while, in 2 patients, the C7 was bridged to the triceps branch of the radial nerve [Figure 5]. In all cases epineural repair was performed using 10/0 nylon under the microscope. Fibrin glue has been available since 2005 and has been added (n=10) to augment the repair whenever possible.

**Table 2:** Distribution of target nerves for grafting from root stumps in the neck

Target nerves	Number of cases
Anterior and posterior divisions of the upper trunk	1
Lateral root of median nerve	11
Median nerve	10
Musculocutaneous nerve	2
Pectoral nerves	2
Radial nerve	2
Lateral cord	4
Suprascapular nerve	1
Axillary nerve	1

### Post-operative care

The limb was immobilized in a stockinet sling or an elbow pouch for 3 weeks after which no immobilization or splinting was used. Shoulder mobilization was restricted to 30° for 2 months after the operation, particularly when intercostals were transferred. Stimulation of the target muscles was advised whenever the patient had access to supervised physiotherapy. The patients were reviewed at 3 and 6 months from the operation and, subsequently, at 6 monthly intervals for any further recovery or worsening of muscle power. Recovery in target muscles was assessed clinically and patient was instructed for strengthening of each muscle as it recovered.

## RESULTS

### Restoration of biceps power [Table 3]

The spinal accessory nerve transfer was used in 21 cases. In 14/21 patients (66%), strength of grade 3 or more was regained in the biceps, while grade 2 power was obtained in 2/21 patients, remaining gained power < grade 2. Intercostals were transferred to musculocutaneous nerve in 59 cases. In 47/59 patients, grade 3 or stronger biceps was regained (77.7%), while in 6 cases, grade 2 biceps was obtained. The successful cases included two patients in whom nerve grafting was necessary. Nerve grafting from proximal C5 stump in the neck to musculocutaneous nerve was performed in seven patients with recovery of grade 4 power in biceps in 5/7 cases (71%). Contralateral C7 transfer was utilized for biceps in six patients. Only two of the six cases recovered grade 4 biceps (33%). Summarizing, grade 3 or stronger biceps was obtained in 68 of the 93 patients using various nerve transfers (73%).

**Table 3:** Results of nerve transfers

Nerve transferred	Number of patients	Recovery of ≥ grade 3 power (%)
Restoration of biceps power		
Spinal accessory	21	14 (66)
Intercostal nerves	59	47 (77.7)
C5 stump	7	5 (71)
Contralateral C7	6	2 (33)
Total	93	68 (73)
Restoration of shoulder abduction		
Spinal accessory	49	43 (89)
Restoration of pectoral muscle power		
Intercostal nerves	8	8 (100)

	**Number of patients**	**Recovery of grade 2 power (no. of patients)**

Restoration of triceps power		
Intercostal nerves	15	12 (80)
Contralateral C7		1

### Restoration of abduction

The spinal accessory nerve was transferred to the suprascapular nerve for restoration of some stability and abduction at the shoulder in 49 cases. Grade 3 function of the supraspinatus was obtained in 43/49 patients (89%). No useful shoulder abduction was seen in remaining six patients. The shoulder abduction regained was limited to 30-45° (n=36) and, in some cases, up to 70-80° (n=7).

### Restoration of triceps

Intercostals were transferred for triceps in 15 cases. Grade 2 recovery in the triceps was considered a success as these patients could not abduct their shoulders above 90°. Grade 2 or stronger triceps was achieved in 12 patients (80%). In one patient, the C7 was bridged to the radial branch to the triceps using sural nerve graft cables [Figure 5] and, in another case, to the radial nerve using a pedicled vascularized ulnar nerve graft. Both cases have shown early contraction of the triceps at 13 months of follow-up. A further improvement with time is expected.

### Restoration of median nerve function

Nerve grafting for the median nerve was performed from the ipsilateral proximal nerve root stumps in 22 patients. Only six of these recovered grade 2 flexion in extrinsic flexors of the fingers and of the wrist (27%). Of these, five patients were younger than 20 years at the time of surgery. Nerve grafting from contralateral C7 to median nerve was performed in 27 cases. Flexion of the fingers was obtained in seven patients (26%). Only two of these patients were older than 20 years, while three were younger than 10 years.

Increasing confidence with various procedures has prompted the senior surgeon (AB) to perform all available transfers at primary surgery. Thus, 18 patients underwent transfer of single nerve (spinal accessory or intercostals for the biceps), while two nerves were transferred in 30 cases, three transfers in 36 cases and four or more nerve targets were dealt with in 9 cases. All cases were operated only once for nerve transfers.

### Secondary operations

Secondary orthopaedic procedures are usually required once the nerve transfers have provided certain motor functions. These include operations to stabilize the shoulder and the wrist and osteotomy of the humerus to change the direction of elbow flexion from the coronal to the sagittal plane. Occassionally, the primary operation has failed to produce elbow flexion while other muscles have got reinnervated. In such cases, the restored muscles (triceps, pectoralis major) are transferred to try and regain elbow flexion.

Shoulder fusion combined with triceps to biceps transfer was done in two cases, while in another patient both the pectoralis major and the triceps were transferred to the biceps. The secondary operations performed were derotation osteotomy of humerus (n=13), wrist fusion (n=14), shoulder fusion (n=7), triceps to biceps transfer (n=3), biceps to FDP transfer (n=2), biceps to ECRB transfer (n=2) and clavicle plating (n=1).

One of the patients in our series operated for flail right upper limb secondary to traumatic brachial plexus injury. He was operated with spinal accessory to suprascapular nerve transfer and intercostal to musculocutaneous and radial nerve transfer. One year post-surgery, he had regained grade 3 power in triceps and grade 4 power in biceps. Since the elbow was stable enough a secondary procedure was planned. The wrist was fused and the biceps was transferred to the flexor digitorum profundus using a fascia lata graft. At 2 year post-surgery, he was able to abduct the shoulder, flex the elbow, and grip the telephone receiver.

## DISCUSSION

The tension at the nerve repair site was the most important detrimental factor for results of nerve suturing and Millesi demonstrated the utility of nerve grafting.^20^ The hurdle of the absence of proximal nerve stumps (because of pre-ganglionic injuries) has been partly overcome with increasing confidence by the use of nerve transfer techniques. The treatment of flail upper limbs demands a logical use of these techniques to achieve maximum benefit in terms of control of the shoulder, elbow, and hand.

The spinal accessory to suprascapular nerve transfer is the usual method employed to restore shoulder stability, abduction (± external rotation) in total palsies. The results reported in the literature have varied from 80% good and fair^21^ to less than 30% fair results.^22^ Terzis **et al**.^23^ (2006) reported 79% good and excellent results in a series of 118 cases of suprascapular nerve reconstruction. However, their series was not restricted to total palsies and included 72 cases in which the axillary nerve was also reconstructed. In our series, the spinal accessory to suprascapular nerve transfer was performed in 49 cases and useful shoulder abduction was achieved in 43 patients. However, external rotation of the shoulder was restored only in patients younger than 20 years. The amount of abduction obtained was poor in the two cases in which a nerve graft was required (one of them recovered external rotation beyond the neutral position but practically no abduction). Nerve grafting from the C5 stump to the suprascapular nerve was done in only one patient with a 30° abduction being obtained. Thus, whenever feasible, direct spinal accessory transfer to suprascapular nerve gives most satisfactory results.

The spinal accessory to musculocutaneous nerve transfer gained popularity from the 1970s through the publications of Kotani **et al** and Allieu **et al**.^24^,^25^ Songcharoen **et al**^26^ have published on a series of 216 patients in which 72.5% cases recovered biceps of grade 3 or stronger. In this series, 14 of 21 patients successfully regained biceps > grade 3 (66.6%). The use of intercostal nerve transfers for the musculocutaneous nerve was initially reported by Seddon.^27^ However, their use became more prevalent following the reports of Nagano and Chuang.^16^,^28^ Nagano reported return of grade 3 and stronger biceps in 56 out of 66 cases (88%) while Chuang showed a success rate of 67%. Chuang did mention that the percentage of success improved with time because of greater familiarity with the procedure and care taken to avoid tension at the repair site, use of three intercostals and with direct repairs (no nerve grafts). In our series, 47 of 59 patients (77%) recovered elbow flexion stronger than grade 3. Nerve grafts were required in three patients because of a second-level rupture of the musculocutaneous nerve discovered during surgery. Two of these regained grade 3 biceps while the third patient had only grade 2 functions. Again poor results were seen with use of nerve grafts.

The intercostals were transferred for triceps function when other neurotizations were available for the biceps. This transfer has been reported by Doi associated with free microvascular transfer of the gracilis muscle for finger flexion.^29^ In this series, the purpose of restoration of triceps is to improve stability of the elbow and to allow the patient to reach out for objects. In the absence of abduction above 90°, even grade 2 recovery of the triceps is sufficient and this was achieved in 80% of cases. In two cases, the triceps could be used to restore elbow flexion as the transfer to the musculocutaneous nerve failed. In another patient, the stability provided by the triceps allowed for fusion of the wrist and transfer of biceps to the flexor digitorum profundus to produce flexion of the fingers. Recently, Oberlin **et al**.^30^ published seven cases treated with the same technique and we present our results of two patients as a part of this cohort study. Gu **et al**. first reported on the possibility of using the C7 root of the intact contralateral brachial plexus as a source of growing axons to reinnervate the hand in cases with all five roots avulsed.^14^ They recommended the use of vascularized ulnar nerve grafts to bridge the long distance to the target nerve. Others have also explored the use of sural nerve grafts.^31^ After the initial excitement about the discovery of a new source of a large number of nerve fibers, the results in reconstruction of flexion of the fingers have been disappointing. Waikakul reported 29% and 20% success rates in restoration of wrist and finger flexion respectively in a series of 96 cases.^15^ Terzis (2008) reported on a series of 56 patients with different target nerves for the contralateral C7.^32^ She showed restoration of biceps in 26% cases, triceps in 43%, and flexion of the wrist and fingers in 38% of cases. In our series, biceps of grade 4 was regained in two of six patients in whom the C7 was connected to the musculocutaneous nerve via sural nerve grafts [Figure 5]. Flexion of the fingers and the wrist was obtained in 7 out of 27 patients (26%). Only two of these were older than 20 years. The lower percentage of success could, perhaps, be explained by the fact that the lateral root of the median nerve was chosen as the target. This is known to have a greater sensory contribution. The preponderance of younger patients in the successful results matches the experience of the other authors.^15^,^32^

Combination of different nerve grafting and nerve transfers in one operation helps these patients regain better function by re-innervation of a larger number of muscles. Selection of the target nerves depends upon the surgeon’s experience and the order of priority. Alnot **et al**. reported on 50 cases.^33^ They followed the traditional method of concentrating on shoulder stability, elbow flexion, and median nerve function. However, they did not use intercostals or contralateral C7 (the latter technique was not published then). Bentolila **et al**. analyzed a series of 57 patients with complete palsy treated by nerve transfer.^11^ Their first priority was elbow flexion and this was achieved in 36 cases (63%). The other targets were extension of the wrist (bypassing the triceps), flexor digitorum superficialis, and deltoid muscles. Shoulder stability was achieved by arthrodesis. However, the report included cases in which two transfers were combined for the biceps (spinal accessory and intercostals). Contralateral C7 was not mentioned as the series referred to patients operated upon till 1991. Strangely, only two root stumps were found in the neck in 29 of their patients. On the other hand, the majority of our patients had avulsions of all five roots (two roots were found only in five patients).

Terzis reported on a series of 204 cases of flail upper limbs.^34^ However, her list included retroclavicular and infraclavicular injuries. There were 20 cases of gunshot wounds and 55 work-related accidents. The order of priority was biceps, triceps, flexor digitorum profundus, and extensor digitorum communis muscles. The plan included at least two major micro-neural and microvascular procedures. Shoulder stabilization depended upon secondary muscle transfers (latissimus dorsi and teres major. Overall, she reported return of biceps in 48% of cases.

In our series, only cases of closed traction injuries of the supraclavicular brachial plexus (root injuries) have been included. All the nerve procedures were performed by single operating surgeon at single surgical setting. The order of priority was the biceps and shoulder stability. In younger patients, an attempt was made to restore some function in the median territory. The overall percentage of elbow flexion restored (73%) compared favorably with those reported earlier. Disappointing results for distal function have prompted to shift to the use of the contralateral C7 for the triceps. This is in concurrence with Doi **et al**.^29^ who now recommend use of contralateral C7 for shoulder function, while retaining the spinal accessory and intercostal nerves for innervation of functioning gracilis transfers that animate the elbow and fingers. Chuang, too, has mentioned a recent shift in preference to retain a nerve transfer for the free gracilis for elbow flexion and finger extension.^35^ We have started to explore these options too and early results look promising. We have already used the technique of Oberlin **et al**.^30^ to achieve acceptable distal function in two patients. The short follow up is one of the drawbacks of our study. A longer follow up will be required not only to assess further improvements especially in cases with median nerve transfers, but also to assess any deterioration in the regained function. The study population is heterogeneous differing in presentation and also in treatment advocated; however important conclusions with respect to effectiveness of nerve reconstruction can be derived.

## CONCLUSION

Acceptable function (restoration of biceps power ≥3) can be obtained in more than two thirds (73%) of these global brachial plexus injuries by using the principles of early exploration and nerve transfer with intense rehabilitation. Whenever feasible an attempt to restore some distal function is worthwhile in selected patients.
